# Screening some pine species from North America and dried zones of western Asia for drought stress tolerance in terms of nutrients status, biochemical and physiological characteristics

**DOI:** 10.3389/fpls.2023.1281688

**Published:** 2023-11-30

**Authors:** Karim Nouri, Ali Nikbakht, Maryam Haghighi, Nematollah Etemadi, Mehdi Rahimmalek, Antoni Szumny

**Affiliations:** ^1^ Department of Horticulture, College of Agriculture, Isfahan University of Technology, Isfahan, Iran; ^2^ Department of Food Chemistry and Biocatalysis, Wrocław University of Environmental and Life Sciences, Wrocław, Poland

**Keywords:** climate change, drought resistance, forest management, pine species urban forestry, water shortage

## Abstract

Drought due to climate change or reduced precipitation is one of the main factors limiting the growth and establishment of plants and is one of the most critical challenges facing humans. To investigate the effect of different levels of drought stress on some pine species, this research was carried out as a factorial experiment using two factors and a completely randomized design. It included five populations of four pine species (*Pinus brutia* Ten. var. eldarica, *P. nigra* Arnold, *P. mugo*, and *P. banksiana* Lamb (including populations 8310055 and 8960049), and three levels of irrigation (100%, 75%, or 50% FC, denoted as normal, mild or intense drought stress, respectively) with three replicates. The findings showed that, photosynthetic pigments, relative water content, visual quality, the content of nutrients, protein content, and fresh and dry weight all decreased significantly when plants were exposed to intense drought stress. However, raised proline levels, electrolyte leakage percentage, soluble sugars levels, and antioxidant enzyme activity. We detected a decline in most growth traits when comparing mild drought stress conditions to normal irrigation, yet acceptable quality seedlings when compared to intense drought stress. Intense drought stress had a substantial impact on many pine seedlings. PCA results showed that among different pine species, the level of resistance to drought is as follows: *P. mugo*> *P. brutia* var. eldarica> *P. nigra*> *P. banksiana* 8310055> *P. banksiana* 8960049. Our novel finding was that, *P. mugo* is a resistant species in arid and semi-arid regions, and *P. banksiana* species, especially its population of 8960049, is sensitive.

## 1 Introduction

Pinus is a genus in the Pinaceae family with more than 100 species that grow in temperate and subtropical climates around the world (Farjon, 2018). Different pine species have different soil and climate requirements, and some are better adapted to acidic soils while others are better adapted to calcareous soils. This allows them to thrive in different regions with varying soil types and climate conditions, such as hot, dry, and semi-arid areas. (Sadegh Kasmaei et al., 2019). Forty-nine species of pine are native to North America. They are very successful in maintaining solid and valuable stands(Gernandt et al., 2005).

Pine’s stress-resistant and pioneering capabilities, as well as its function in supporting the long-term growth of late-successional hardwoods, provide a theoretical basis for its application (Rincón et al., 2021). Many pine species add excellent aesthetic features to large parks and gardens, while dwarf species are better suited to smaller places (Robinson, 2017). Iran country is located in an arid region, and some pine trees have adapted to its climate (Sohrabi et al., 2016), so three of these pines, in terms of resistance to drought stress and the ability to plant in arid and semi-arid areas with species of North America, were compared, which these species include:

The dwarf mountain pine (*Pinus mugo* Turra) is a species native to Central and Southern Europe that shows adequate resistance to biotic and abiotic stressors (Wielgolaski et al., 2017). This pine species can adapt to a variety of soil conditions, including dry sandy areas, alkaline and acidic layers, and drought (Bičárová et al., 2019).

Jackpine (*Pinus banksiana*) is small to medium-sized coniferous tree that this species thrives in soils that are less suitable and drier than those required by other native species in its habitat, and it can grow in extremely dry sandy or gravelly soils where other species would struggle to survive (Xu, 2018).

Black Pine (*Pinus nigra* J.F. Arnold) is an extensively planted ornamental plant in Europe, the United States, and other regions of the world (Novotný et al., 2018). It is also known as European black pine, Austrian pine, or Crimean pine. This tree can withstand a variety of adverse environmental circumstances, including extreme heat and cold, drought stress, and urban pollution. This pine thrives in both arid and wet environments, with a high tolerance to temperature changes and resistance to drought and wind (Martin-Benito et al., 2013).

*Pinus brutia* var. eldarica is endemic to Azerbaijan and is somewhat unique among pinus species in its tolerance to environmental stress. It adapts well to high-pH soils and has been described as a drought-tolerant tree (Famil, 2021).

Both urban and forest tree populations have been subjected to new selection pressures in recent decades, and they may not be well established after planting and may perish (Trugman et al., 2021). The initial years are the most critical for establishing tree species because seedlings are vulnerable to resource constraints, such as drought, salt, low soil fertility, pests and diseases, and pollution (Askari-Khorasgani and Pessarakli, 2019). In addition, climate change and global warming exacerbate the impact of environmental stresses on plants (Santoyo et al., 2017). Plant growth morphology, growth physiology, gene expression, and cellular metabolism are all affected by drought stress (Farooq et al., 2009). Different environmental stresses, such as drought, have resulted in the production of a variety of damaging oxygen species, some of which act as free radicals in plant cells (Gill and Tuteja, 2010). If the plant’s defense system is ineffective, free radicals start with harmful activities, including chlorophyll degradation, lipid peroxidation, or protein oxidation (Halliwell, 2006). The response of seedlings to a water shortage is influenced by a variety of parameters, including growth stage, stress intensity, stress duration, and plant genetics. Stunted development, slowed photosynthesis, accelerated leaf aging, and other symptoms have been seen in plants as a result of water shortages (Jain et al., 2019). A number of physiological and biochemical processes enable plants to respond to drought stress. Physiological processes include the buildup of compatible osmolytes such as proline and soluble carbohydrates, the production of antioxidant molecules such as ascorbate and glutathione, and the increase of antioxidant enzyme activity (Gupta and Huang, 2014). To combat drought stress, plants expand complicated growth, physiological, and biochemical systems, categorized into escape, avoidance, and/or drought tolerance (Salehi-Lisar and Bakhshayeshan-Agdam, 2016). Also, Several hydrophilic (ascorbate and glutathione) and lipophilic (alpha-tocopherol) antioxidants, as well as enzymes (catalase, superoxide dismutase, and guaiacol peroxidase), are found in the antioxidant defense system (Ighodaro and Akinloye, 2018). These enzymes have the ability to mitigate the effects of ROS. As a result, determining each plant’s drought resistance mechanisms is a crucial topic in environmental and survival research (Laxa et al., 2019). Because pine species are prevalent and fundamental parts of many forests and urban landscapes (Frank et al., 2013), extensive studies are needed to help in the selection of the most drought-resistant species for future urban and forest projects. In light of this, the purpose of this study was to examine and compare the morphological and physiological responses of the most significant drought-resistant pine species to varying levels of drought stress. In addition, it is anticipated that the outcomes of this study will enhance our understanding of the mechanisms underpinning drought resistance responses in the species, enabling us to establish more effective management strategies and, eventually, to choose suitable candidates that can tolerate environmental challenges and prevent large-scale afforestation failure.

## 2 Materials and methods

### 2.1 Experimental site and soil properties

A three-year experiment was conducted at Isfahan University of Technology’s (IUT) horticulture department’s campus facilities (32°39′ N, 51°40′ E; 1590 m) in Isfahan, Iran, from 2019 to 2021. The seeds extracted from cones before stratifying them in the dark at 5°C for 4 weeks to meet the chilling requirement. Then, seeds of various species were put in a tray and kept at 25°C in a greenhouse until they germinated. Next, germinants transplanted to plastic pots (dimensions: 32 cm diameter top, 24.5 cm diameter base, and 31 cm pot depth), filled with an autoclaved (120°C for 2 h) soil:sand (2:1, v/v) mixture. Furthermore, to supply minimum nutrient levels during the experiment, we fertilized seedlings with 2 g L^−1^ of a commercial fertilizer (20-5-10 N-P-K, 12.8% S, and 1.3% MgO) (NovaTec Solub, Compo, Germany) at transplanting, before the start of irrigation regimesThe original soil had a clay loam texture (28 percent clay, 42 percent silt, and 30 percent sand), a pH of 7.7, an electrical conductivity of 1.02 dS m^-1^, and 35.1 percent (CaCO_3_) limestone. The soil contained 8.27 g. kg^−1^ organic matter, 30 mg.kg^−1^ N (total), 6.2 mg.kg^−1^ available P, 120 mg.kg^−1^ K (exchangeable), 96 mg.kg^−1^ Mg (available or extractable), 8.5 mg. kg^−1^ Fe, and 0.7 mg.kg^−1^ Zn.

### 2.2 Experimental design and treatments

This study was conducted as a factorial experiment based on a completely randomized design (CRD) with two factors: five populations of four pine species and three levels of irrigation (100 (normal), 75 (mild drought stress), and 50 (intense drought stress) percent of field capacity), with three replicates and six seedlings in each replication. Seeds of species (*P. brutia* var. eldarica, *P. nigra*, *P. mugo*) pine were collected from the Forest of IUT. Seeds from a single tree were obtained to minimize genetic diversity between seedlings of each species. *P. banksiana* seeds were collected from two distinct regions of Canada [(populations 8310055 and 8960049 from Canterbury and Calling Lake, respectively (**Table 1**)].

**Table 1:** Origin of seed collection of pine species used in the experiment.

Species	Origin of seed collection
*Pinus brutia Ten.* var. *eldarica*	Forest of Isfahan University of Technology, Isfahan, Iran
*Pinus mugo*	Forest of Isfahan University of Technology, Isfahan, Iran
*Pinus nigra* Arnold	Forest of Isfahan University of Technology, Isfahan, Iran
*Pinus banksiana* Lamb. population 8310055	Canturbury, Ontario, Canada
*Pinus banksiana* Lamb. population 8 960049	Calling Lake, Alberta, Canada

Seedlings in pots were adequately watered for three months to assure their establishment, before being subjected to drought stress for the next six months. For the irrigation regime, 3 months after the transplantation we used either 100%, 75%, or 50% field capacity, denoted as normal, mild or intense drought stress, respectively. As a basis for irrigation, we measured matrix potential of media in two pots per treatment/control. Specifically, we monitored media potential using a tensiometer (Soil moisture 2710 ARL, Soil moisture Equipment Corp., USA) placed at a depth of 15 cm. On each monitoring date, we also determined media moisture on a weight basis. After determinations were finished, when the soil water potential reached -10 kPa, the seedlings were irrigated to pot capacity, then mild and intense drought stress treatments were applied; seedlings received 75 percent and 50 percent of the water used for normal condition (control treatment), respectively.

### 2.3 Fresh and dry weight of shoot

Each treatment’s seedlings of pine species were harvested at the end of the drought stress period. The aerial part was made up of the needles and stem, which were weighed by laboratory scale (M6202i, accuracy =0.01gr, Bel Engineering, Monza and Brianza, Italy) and used to determine the fresh weight (FW) of the shoot. After drying in an oven at 78°C for 48 hours, the samples were weighed again to obtain the dry weight (DW) (Cho et al., 2007).

### 2.4 Chlorophyll and carotenoid contents

To determine the amount of chlorophyll and carotenoids, Lichtenthaler’s technique was used (Lichtenthaler, 1987). Pigments were extracted from one gram of fresh leaf in liquid nitrogen, then homogenized with 10 mL absolute acetone and centrifuged for 10 minutes at 5000 g. The photosynthetic pigments absorbance was measured spectrophotometrically at 645 and 662 nm for chlorophyll a and b, respectively, and the carotenoid at 470 nm (UV-160A, Shimadzu, Tokyo, Japan). Finally, the concentrations of the extracted pigments were calculated using equations described by Lichtenthaler (1987).

### 2.5 Relative water content

Using a modified version of the Ritchie et al. approach, the relative water content (RWC) was obtained (Ritchie et al., 1990). In this approach, one gram of needles from each sample was weighed (W1). Then, the samples were drenched in 10 mL deionized water for 24 hours at 4°C and weighed to determine the turgid weight (W2). Next, the samples were dried for 24 hours at 80°C, and the dry weight was recorded (W3). Finally, RWC was calculated using the following equation:


1) RWC=(fresh weight–dry weight)/(turgid weight–dry weight) × 100

### 2.6 Electrolyte leakage

The Lutts et al. (1996) method was used to investigate electrolyte leakage. Randomly chosen freshly needles (1 gr) were rinsed three times to get rid of any surface contaminates. before being immersed in 10 mL of deionized water. The electrical conductivity (EC) of the bathing solution (EC1) was then measured after the samples were incubated at room temperature on a shaker (150 rpm) for 22 h. The samples were then boiled for 2 hour at 95°C, cooled, and the final EC (EC2) was measured. The equation of (EC1/EC2) × 100 was used to determine EL.

### 2.7 Proline and soluble sugars content

The leaves of the plants were examined for proline content. The Bates et al. (1973) process was followed with small modifications. First, 0.2 g of fresh leaf sample was homogenized in 10 ml of three percent (w/v) sulfosalicylic acid. Then, one ml of this homogenous solution was reacted for 60 minutes at 100°C with one ml of a mixture of glacial acetic acid and orthophosphoric acid (6 M) (3:2, v/v) and 25 mg acid ninhydrin. After cooling the reaction in an ice bath, two ml of toluene was used to extract the product. Using a spectrophotometer (UV-160A, Shimadzu, Tokyo, Japan), the upper phase absorbance was measured during the determination’s follow-up phase at 520 nm. Based on a standard curve created using L-proline, the free proline amount in each fresh tissue gram was calculated and expressed.The Anthrone-sulfuric acid method was used to determine the content of soluble sugars, which was measured using a standard sugars solution and expressed as mg.g^-1^ FW of samples. Under acidic and boiling circumstances, sugars react with the anthrone reagent to produce a blue-green color complex that can be quantified colorimetrically at 620 nm (Grandy et al., 2000).

### 2.8 Antioxidant enzymes activity

100 mg of fresh leaves samples were combined with 1 mL buffer (1 percent polyvinylpyrrolidone, 0.5 percent triton X100, and 100 mM K-phosphate buffer, pH 7.0) to determine the activity of specific antioxidant enzymes. 20 minutes were spent centrifuging the homogenate solution at 15,000 g and 4°C. Following that, the antioxidant enzymes’ activity or content was determined as follows:

Catalase (CAT) activity was determined using a modified version of Aebi’s method (Aebi, 1984). H_2_O_2_ disintegration was detected spectrophotometrically at 240 nm after the indicated activity. 2.95 mL of reaction buffer [50 mM K-phosphate buffer (pH 7.0)], and 15 mM H_2_O_2_ was mixed with 0.05 mL of enzyme extract for this assay. The specific activity of the CAT enzyme was determined by dividing the amount of CAT activity on the protein isolated using the Bradford technique (Bradford, 1976). The results were expressed in terms of relative enzyme activity per milligram of protein.

Ascorbate peroxidase (APX) activity was determined using the method suggested by Nakano and Asada (1981) with some modifications. First, 2.95 mL from the reaction buffer, 5 mM ascorbate (AsA), 0.5 mM H_2_O_2_, and 0.05 mL of enzyme extract was begun by the addition of H_2_O_2_. Then, the activity was measured by observing the reduction in the absorbance at 290 nm for 2 min using a spectrophotometer (UV-160A, Shimadzu, Tokyo, Japan).

The activity of guaiacol peroxidase (GPX) was evaluated using H_2_O_2_ as a substrate, as described by Rao et al. (1996). NADPH oxidation was monitored for 2 minutes at 470 nm, and activity was calculated by dividing the quantity of GPX activity by the total protein content.

The activity of superoxide dismutase was determined using a technique developed by Giannopolitis and Ries (1977). First, 50 μl of the enzyme extract was added to 3 ml of the reaction buffer, 75 nm EDTA, 13 mM methionine, and 63 μm nitroblue tetrazolium, followed by 1.3 μm riboflavin. The materials were exposed to light for 15 minutes before being measured using a spectrophotometer (UV-160A, Shimadzu, Tokyo, Japan) at 560 nm. 50% color reduction was considered for one unit (U) of SOD activity; the activity was shown as units per mg of the total protein content. The control reaction mixture did not contain any crude enzyme extract, while the blank reaction mixture had identical ingredients, but was kept in the dark.

Using the Bradford method (Bradford, 1976) and bovine serum albumin as a reference, the protein content of the enzyme extracts was assessed.

### 2.9 Nutrients content

The mineral content of plant leaves was determined at the end of the experiment. The oven drying at 70°C for 48 hours produced dried leaves. After 550°C, 5.5 hours of dry ashing, 2 M HCl was used to remove Ca, K, Fe, and Zn from the samples (Aalipour et al., 2021). The Kjeldahl method was used to measure nitrogen following digestion with sulfuric acid, salicylic acid, and hydrogen peroxide (Eaton et al., 1995). Using an atomic absorption device (AA670 Shimadzu, Kyoto, Japan), iron and zinc were measured (AOAC, 2006). Potassium concentration was determined using a photoelectric flame photometer (PFP7). The Cottenie method was utilized to detect phosphorus calorimetrically with a wavelength spectrophotometer at 880 nm (UV-160A, Shimadzu, Tokyo, Japan) (Cottenie, 1980).

### 2.10 Visual quality

To assess the impact of treatments on seedling visual quality, color, and needle chlorosis rates were divided into three categories: good (67-100 percent green without chlorosis and yellowing), medium (33-37 percent of entirely green needles with chlorosis and yellowing), and poor (0-33 completely green needles with burns and yellowing) and then ranked (3 = good, 2 = medium, 1 = weak) (Rodriguez and Miller, 2000).

### 2.11 Statistical analysis

The parameters’ values were subjected to a two-way analysis of variance (ANOVA), and the mean differences were compared using the LSD Test. Each data point represented the average of three replicates (each replicate included six seedlings). The statistical analysis was performed using SAS statistical software version 9.2 (SAS Institute, Cary, NC, USA).

## 3 Results

### 3.1 Fresh and dry weight of shoots

The analysis of the variance (**Table 2**) indicated that the irrigation levels and pine species affected FW and DW. Drought stress resulted in a considerable decrease in FW and DW (P<0.01). The mean comparison for FW and DW revealed that *P. mugo* species had the greatest FW and DW, while *P. banksiana* species population 8960049 had the least DW (**Table 3**). The results showed that under intense drought stress, FW (by 30 percent) and DW (by 24 percent) reduced compared to the normal treatment (**Table 4**).

**Table 2:** Analysis of variance for fresh weight, dry weight, chlorophyll a, chlorophyll b, total chlorophyll, and carotenoid contents of five pine species (*P. brutia* var. eldarica, *P. mugo, P. nigra, P. banksiana* 1*, P. banksiana* 2) under three drought stress treatments (normal, mild, and intense drought stress).

S.V	df	Mean square					
		Fresh weight	Dry weight	Chlorophyll a	Chlorophyll b	Total chlorophyll	Carotenoid
Rep	2	0.286 ^ns^	0.184 ^ns^	0.0034^ns^	0.001^ns^	0.018^ns^	0.002^ns^
Species	4	2.69^**^	0.321^**^	0.0211^**^	0.009^**^	0.056^**^	0.003^**^
Stress	2	14.3^**^	2.72^**^	0.5^**^	0.053^**^	0.880^**^	0.14^**^
Species × Stress	8	0.67 ^ns^	0.044^ns^	0.0031^ns^	0.001^ns^	0.003^ns^	0.001^*^
Error	28	0.183	0.63	0.02	0.013	0.037	0.0004
CV		6.36	12.16	6.17	10.89	5.27	6.57

*: Significant at the 0.05 probability level, **: Significant at the 0.01 probability level, ns, non-significant.

S.V, source of variance; df, degree of freedom; Rep, replication; CV, coefficient of variance.

Normal, 100% field capacity (FC); Mild, 75% FC; Intense, 50% FC.

**Table 3:** Mean comparison of **f**resh weight, dry weight, chlorophyll a, chlorophyll b, total chlorophyll, and carotenoid contents for five pine species (*P. brutia* var. eldarica, *P. mugo, P. nigra, P. banksiana* 1*, P. banksiana* 2).

Species	Fresh weight (gr)	Dry weight (gr)	Chlorophyll a (mg.gr^-1^ fw)	Chlorophyll b (mg.gr^-1^ fw)	Chlorophyll Total (mg.gr^-1^ fw)	Carotenoid (mg.gr^-1^ fw)
*P. brutia.* var. eldarica	6.79 ± 0.79^b^	2.07 ± 0.33^bc^	0.758 ± 0.14^b^	0.300 ± 0.07^a^	1.05 ± 0.21^b^	0.352 ± 0.07^a^
*P. mugo*	7.62 ± 0.99^a^	3.35 ± 0.43^a^	0.808 ± 0.13^a^	0.309 ± 0.05^a^	1.11 ± 0.18^a^	0.361 ± 0.07^a^
*P. nigra*	6.65 ± 0.98^bc^	2.13 ± 0.60^ab^	0.726 ± 0.15^bc^	0.254 ± 0.04^b^	0.98 ± 0.18^c^	0.319 ± 0.08^b^
*P. banksiana*1	6.33 ± 0.88^c^	1.92 ± 0.33^bc^	0.701 ± 0.16^c^	0.250 ± 0.05^b^	0.92 ± 0.21^c^	0.323 ± 0.09^b^
*P. banksiana*2	6.24 ± 0.79^c^	1.87 ± 0.31^c^	0.687 ± 0.17^c^	0.237 ± 0.06^b^	0.93 ± 0.22^c^	0.329 ± 0.08^b^

In each column, means followed by a common letter are not significantly different according to the LSD test at an alpha level of 0.05.

P. banksiana1: P. banksiana population 8310055, P. banksiana2: P. banksiana population 8960049.

**Table 4:** Mean comparison of fresh weight, dry weight, chlorophyll a, chlorophyll b, total chlorophyll, and carotenoid contents under three drought stress treatments (normal, mild, and intense drought stress).

Drought stress levels	Fresh weight (gr^-^)	Dry weight (gr)	Chlorophyll a (mg.gr^-1^ fw)	Chlorophyll b (mg.gr^-1^ fw)	Chlorophyll Total (mg.gr^-1^ fw)	Carotenoid (mg.gr^-1^ fw)
Normal	2.56 ± 0.69^a^	7.77 ± 0.29^a^	0.897 ± 0.04^a^	0.328 ± 0.04^a^	1.225 ± 0.06^a^	0.433 ± 0.02^a^
Mild drought stress	1.86 ± 0.52^b^	6.57 ± 0.34^b^	0.774 ± 0.05^b^	0.274 ± 0.04^b^	1.049 ± 0.09^b^	0.338 ± 0.02^b^
Intense drought stress	1.78 ± 0.60^b^	5.83 ± 0.21^c^	0.538 ± 0.07^c^	0.208 ± 0.03^c^	0.746 ± 0.09^c^	0.240 ± 0.03^c^

In each column, means followed by a common letter are not significantly different according to the LSD test at an alpha level of 0.05.

Normal: 100% field capacity (FC), Mild: 75% FC, Intense: 50% FC.

### 3.2 Photosynthetic pigments

The results (**Table 2**) showed both irrigation levels and pine species significantly affected chlorophyll content (P<0.01). The mean comparison for chlorophyll a, b, and total (Chl. a, b, T), as well as carotenoid content (CAR) between pine species revealed that *P. mugo* species had the highest concentrations of Chl. a, b, T, and CAR, while population 8960049 of *P. banksiana* species had the lowest concentration of Chl. a, b, and population 8310055 of *P. banksiana* species had the lowest concentration of total chlorophyll (**Table 3**). Furthermore, the mean comparison for photosynthetic pigments demonstrated that under intense drought stress, the concentration of Chl. a (by 40%), b (by 37%), T (by 39%), and CAR (by 44%) reduced compared to the normal treatment (**Table 4**). For CAR pigment, the interaction effect of drought stress and pine species treatment revealed that the highest content of CAR was found in *P. brutia* var. eldarica species under drought stress at 75 percent of field capacity, and the lowest content was found in population 8960049 of *P. banksiana* species under drought stress at 75 percent of field capacity (**Table 5**).

**Table 5:** Interaction effect of drought stress treatments (normal, mild, and intense drought stress) on five pine species (*P. brutia* var. eldarica, *P. mugo, P. nigra, P. banksiana* 1*, P. banksiana* 2), for carotenoid pigment.

Drought stress levels	Species	Carotenoid (mg.gr^-1^ fw)
Normal	*P. brutia.* var. eldarica	0.440 ± 0.03^ab^
	*P*. *mugo*	0.425 ± 0.02^b^
	*P*. *nigra*	0.328 ± 0.02^cde^
	*P*. *banksiana*1	0.357 ± 0.02^c^
	*P*. *banksiana*2	0.230 ± 0.02^fg^
Mild drought stress	*P. brutia.* var. eldarica	0.465 ± 0.02^a^
	*P*. *mugo*	0.417 ± 0.02^b^
	*P*. *nigra*	0.307 ± 0.03^de^
	*P*. *banksiana*1	0.254 ± 0.02^f^
	*P*. *banksiana*2	0.210 ± 0.02^gh^
Intense drought stress	*P. brutia.* var. eldarica	0.420 ± 0.03^b^
	*P*. *mugo*	0.363 ± 0.02^c^
	*P*. *nigra*	0.334 ± 0.01^cd^
	*P*. *banksiana*1	0.292 ± 0.01^e^
	*P*. *banksiana*2	0.213 ± 0.02^g^

In each column, means followed by a common letter are not significantly different according to the LSD test at an alpha level of 0.05.

P. banksiana1: P. banksiana population 8310055, P. banksiana2: P. banksiana population 8960049.

Normal: 100% field capacity (FC), Mild: 75% FC, Intense: 50% FC.

### 3.3 Relative water content

The influence of irrigation levels and pine species on the RWC was significant (P<0.01), according to the analysis of variance (**Table 6**). The most RWC was found in *P. mugo* and *P. brutia* var. eldarica species, according to the mean comparison of RWC. The lowest content was found in population 8310055 of the *P. banksiana* (**Table 7**). The RWC under intense drought stressreduced (by 20%) as compared to the normal irrigation treatment (**Table 8**).

**Table 6:** Analysis of variance for relative water content (RWC), electrolyte leakage, proline content, soluble sugars, and visual quality of five pine species (*P. brutia* var. eldarica, *P. mugo, P. nigra, P. banksiana* 1*, P. banksiana* 2) under three drought stress treatments (normal, mild, and intense drought stress).

S.V	df	Mean square				
		RWC	Electrolyte leakage	Proline	Soluble sugars	Visual quality
Rep	2	6.48^ns^	12.92^ns^	0.41^ns^	0.55 ^ns^	**0.014^ns^**
Species	4	26.8^**^	44.1^**^	0.38^**^	7.63^**^	0.26^**^
Stress	2	1170^**^	493^**^	17.9^**^	519 ^**^	4.65^**^
Species × Stress	8	6.62^ns^	2.69^ns^	0.005^ns^	10.5 ^**^	**0.21^ns^**
Error	28	7.29	10.66	0.053	1.87	**0.030**
CV		8.25	10.29	7.80	3.03	**7.85**

*: Significant at the 0.05 probability level, **: Significant at the 0.01 probability level, ns, non-significant.

S.V, source of variance; df, degree of freedom; Rep, replication; CV, coefficient of variance.

Normal: 100% field capacity (FC), Mild: 75% FC, Intense: 50% FC.

**Table 7:** Mean comparison of relative water content (RWC), electrolyte leakage, proline content, soluble sugars, and visual quality for five pine species (*P. brutia* var. eldarica, *P. mugo, P. nigra, P. banksiana* 1*, P. banksiana* 2).

Species	RWC (%)	Electrolyte leakage (%)	Proline (mg.gr^-1^ fw)	Soluble sugars (mg.gr^-1^ fw)	Visual quality
*P. brutia.* var. eldarica	78.77 ± 8.02^a^	29.77 ± 5.33^b^	2.79 ± 0.93^b^	44.6 ± 5.07^bc^	2.35 ± 0.48^ab^
*P. mugo*	79. 44 ± 6.44^a^	29.11 ± 5.25^c^	2.56 ± 0.88^c^	44.1 ± 6.13^c^	2.38 ± 0.43^a^
*P. nigra*	77.16 ± 6.83^ab^	32.01 ± 5.61^ab^	2.95 ± 0.93^ab^	45.8 ± 4.60^ab^	2.21 ± 0.47^bc^
*P. banksiana*1	75.32 ± 7.76^b^	33.83 ± 5.54^a^	3.06 ± 0.91^a^	46.2 ± 4.47^a^	2.04 ± 0.49^dc^
*P. banksiana*2	76.16 ± 8.78^b^	33.83 ± 5.43^a^	3.04 ± 0.91^a^	44.6 ± 5.12^bc^	2.02 ± 0.50^d^

In each column, means followed by a common letter are not significantly different according to the LSD test at an alpha level of 0.05.

P. banksiana1: P. banksiana population 8310055, P. banksiana2: P. banksiana population 8960049.

**Table 8:** Mean comparison of relative water content (RWC), electrolyte leakage, proline content, soluble sugars, and visual quality under three drought stress treatments (normal, mild, and intense drought stress).

Drought stress levels	RWC (%)	Electrolyte leakage (%)	Proline (mg.gr^-1^ fw)	Soluble sugars (mg.gr^-1^ fw)	Visual quality
Normal	86.8 ± 2.49^a^	25.6 ± 2.94	1.77 ± 0.23^c^	38.7 ± 1.88^c^	2.71 ± 0.17^a^
Mild drought stress	75.8 ± 3.36^b^	32.4 ± 3.66^b^	2.91 ± 0.30^b^	46.1 ± 1.96^b^	2.28 ± 0.19^b^
Intense drought stress	69.40 ± 2.75^c^	37.1 ± 3.56^a^	3.96 ± 0.27^a^	50.3 ± 1.94^a^	1.61 ± 0.26^c^

In each column, means followed by a common letter are not significantly different according to the LSD test at an alpha level of 0.05.

Normal: 100% field capacity (FC), Mild: 75% FC, Intense: 50% FC.

### 3.4 Electrolyte leakage

Electrolyte leakage was influenced by irrigation levels and pine species (P<0.01), according to the findings (**Table 6**). The mean comparison for EL under pine species revealed that *P. mugo* species had the least EL, whereas populations 8310055 and 8960049 of *P. banksiana* species had the greatest (**Table 7**). Furthermore, the intense drought stress boosted EL (by 44%) as compared to the normal irrigation (**Table 8**).

### 3.5 Proline content

The influence of irrigation levels and pine species on proline content was significant (P<0.01), according to the analysis of variance (**Table 6**). The mean comparison for proline revealed that *P. banksiana* species population 8310055 had the highest proline content, while *P. mugo* had the lowest (**Table 7**). Furthermore, the effect of irrigation levels on proline showed that intense drought stress led to an increase in proline content (21%) compared to the normal treatment (**Table 8**).

### 3.6 Soluble sugars

The influence of pine species and irrigation levels on soluble sugars was significant (P<0.01) according to the analysis of variance (**Table 6**). According to the findings*, P. banksiana* population 8310055 had the highest concentration of soluble sugars, whereas *P. brutia* var. eldarica and *P. mugo* had the lowest level (**Table 7**). Additionally, soluble sugar content increased by 123 percent at intense drought stress (**Table 8**). The maximum soluble sugars content was found in *P. banksiana* population 8960049 under intense drought stress, while the least content was found in *P. brutia* var. eldarica species under mild drought stress, according to an interaction effect study between drought stress and pine species treatment for soluble sugars (**Table 9**).

**Table 9:** Interaction effect of drought stress treatments (normal, mild, and intense drought stress) on five pine species (*P. brutia* var. eldarica, *P. mugo, P. nigra, P. banksiana* 1*, P. banksiana* 2), for soluble sugars trait.

Drought stress levels	Species	Soluble sugars (mg.gr^-1^ fw)
Normal	*P. brutia.* var. eldarica	37.9 ± 0.76^hi^
	*P*. *mugo*	40.6 ± 0.76^fg^
	*P*. *nigra*	47.8 ± 0.46^cde^
	*P*. *banksiana*1	42.5 ± 0.50^f^
	*P*. *banksiana*2	50.5 ± 2.20^ab^
Mild drought stress	*P. brutia.* var. eldarica	35.6 ± 0.57^i^
	*P*. *mugo*	39.7 ± 0.55^gh^
	*P*. *nigra*	47.2 ± 0.34^8^
	*P*. *banksiana*1	49.9 ± 0.90^e^
	*P*. *banksiana*2	50.8 ± 2.84^ab^
Intense drought stress	*P. brutia.* var. eldarica	39.7 ± 0.76^gh^
	*P*. *mugo*	46.0 ± 0.05^e^
	*P*. *nigra*	47.3 ± 1.15^de^
	*P*. *banksiana*1	48.9 ± 2.58^bcd^
	*P*. *banksiana*2	51.6 ± 1.44^a^

In each column, means followed by a common letter are not significantly different according to the LSD test at an alpha level of 0.05.

P. banksiana1: P. banksiana population 8310055, P. banksiana2: P. banksiana population 8960049.

Normal: 100% field capacity (FC), Mild: 75% FC, Intense: 50% FC.

### 3.7 Activity of antioxidant enzymes and protein content

The results of the analysis of variance (**Table 10**) showed that the activity of the antioxidant enzymes (CAT, APX, GPX, and SOD) was significantly affected by the pine species and the severity of the drought stress (P<0.01). According to the findings, the antioxidant enzyme activity was highest in population 8960049 of the *P. banksiana* species (**Table 11**). Under intense stress raised the activity of CAT, APX, GPX, and SOD antioxidant enzymes by 77%, 64%, 29%, and 29%, respectively, but decreased protein content by 20%. (**Table 12**).

**Table 10:** Analysis of variance for catalase, ascorbate peroxidase, guaiacol peroxidase, superoxide dismutase activities and protein content of five pine species (*P. brutia* var. eldarica, *P. mugo, P. nigra, P. banksiana* 1*, P. banksiana* 2).under three drought stress treatments (normal, mild, and intense drought stress).

S.V	df	Mean square				
		Catalase	Ascorbate peroxidase	Guaiacol peroxidase	Superoxide dismutase	Protein
Rep	2	14.88^ns^	39.33^ns^	5.40^ns^	93^ns^	0.001^ns^
Species	4	28.4^**^	89.68^*^	107 ^*^	1340^*^	0.047^*^
Stress	2	1350^**^	10464^**^	25011^**^	199545^**^	0.541^**^
Species × Stress	8	1.40^ns^	1.78^ns^	3.75^ns^	18.41 ^ns^	0.056^**^
Error	28	3.75	26.11	34	11.9	0.016
CV		5.88	5.48	3.49	1.61	13

*: Significant at the 0.05 probability level, **: Significant at the 0.01 probability level, ns: non-significant.

S.V: source of variance, df: degree of freedom, Rep: replication, CV: coefficient of variance.

Normal: 100% field capacity (FC), Mild: 75% FC, Intense: 50% FC.

**Table 11:** Mean comparison of catalase, ascorbate peroxidase, guaiacol peroxidase, superoxide dismutase activities and protein content for five pine species (*P. brutia* var. eldarica, *P. mugo, P. nigra, P. banksiana* 1*, P. banksiana* 2).

Species	Catalase (μmol/min/mg protein)	Ascorbate peroxidase (μmol/min/mg protein)	Guaiacol peroxidase (μmol/min/mg protein)	Superoxide dismutase (U/mg protein)	Protein (mg/gr^-1^fw)
*P. brutia.* var. eldarica	31.3 ± 8.45^b^	91 ± 21.21^b^	166 ± 33.70^b^	623 ± 66.21^c^	1.79 ± 0.24^ab^
*P. mugo*	31.4 ± 7.14^b^	89 ± 21.08^c^	161 ± 32.30^c^	618 ± 67.59^d^	1.68 ± 0.14^b^
*P. nigra*	32.4 ± 7.65^b^	92 ± 21.52^b^	167 ± 33.88^b^	627 ± 64.85^b^	1.85 ± 0.20^a^
*P. banksiana*1	34.47.90 ± ^a^	95 ± 22.13 ^a^	169 ± 33.96^a^	630 ± 68.13^b^	1.80 ± 0.22^ab^
*P. banksiana*2	35.2 ± 8.30^a^	97 ± 22.33 ^a^	169 ± 33.34^a^	634 ± 66.5 ^a^	1.86 ± 0.22^a^

In each column, means followed by a common letter are not significantly different according to the LSD test at an alpha level of 0.05.

P. banksiana1: P. banksiana population 8310055, P. banksiana2: P. banksiana population 8960049.

**Table 12:** Mean comparison of catalase, ascorbate peroxidase, guaiacol peroxidase, superoxide dismutase activities and protein content under three drought stress treatments (normal, mild, and intense drought stress).

Drought stress levels	Catalase (μmol/min/mg protein)	Ascorbate peroxidase (μmol/min/mg protein)	Guaiacol peroxidase (μmol/min/mg protein)	Superoxide dismutase (U/mg protein))	Protein (mg/gr^-1^fw)
Normal	23.5 ± 2.21^c^	73 ± 3.38^c^	125 ± 3.31^c^	550 ± 6.41^c^	87 ± 0.16^a^
Mild drought stress	34.1 ± 2.14^b^	84 ± 2.96^b^	1674.32 ± ^b^	618 ± 6.98^b^	76 ± 0.14^b^
Intense drought stress	41.8 ± 2.35^a^	123 ± 4.16^a^	207 ± 4.48^a^	712 ± 5.65 ^a^	69 ± 0.15^c^

In each column, means followed by a common letter are not significantly different according to the LSD test at an alpha level of 0.05.

Normal: 100% field capacity (FC), Mild: 75% FC, Intense: 50% FC.

The interaction between drought stress and pine species treatment for soluble protein content revealed that *P. banksiana* population 8960049 underwent the most intense drought stress and had the highest level of soluble protein content. While, under drought stress, the minimal content of this trait was attained at mild drought stress in the *P. brutia* var. eldarica species (**Table 13**).

**Table 13:** Interaction effect of drought stress treatments (normal, mild, and intense drought stress) on five pine species (*P. brutia* var. eldarica, *P. mugo, P. nigra, P. banksiana* 1*, P. banksiana* 2), for protein trait.

Drought stress levels	Species	Protein (mg.gr^-1^ fw)
Normal	*P. brutia.* var. eldarica	1.53 ± 0.01^g^
	*P*. *mugo*	1.62 ± 0.06^defg^
	*P*. *nigra*	1.65 ± 0.06^defg^
	*P*. *banksiana*1	1.82 ± 0.03^cde^
	*P*. *banksiana*2	2.01 ± 0.16^abc^
Mild drought stress	*P. brutia.* var. eldarica	1.52 ± 0.04^g^
	*P*. *mugo*	1.63 ± 0.07^defg^
	*P*. *nigra*	1.5 ± 0.099^fg^
	*P*. *banksiana*1	2.04 ± 0.05^ab^
	*P*. *banksiana*2	2.05 ± 0.22^ab^
Intense drought stress	*P. brutia.* var. eldarica	1.96 ± 0.04^abc^
	*P*. *mugo*	1.45 ± 0.27^cde^
	*P*. *nigra*	1.75 ± 0.13^def^
	*P*. *banksiana*1	1.82 ± 0.05^cde^
	*P*. *banksiana*2	2.13 ± 0.15^a^

In each column, means followed by a common letter are not significantly different according to the LSD test at an alpha level of 0.05.

P. banksiana1: P. banksiana population 8310055, P. banksiana2: P. banksiana population 8960049.

Normal: 100% field capacity (FC), Mild: 75% FC, Intense: 50% FC.

### 3.8 Nutrients content

Based on the results of the analysis of variance (**Table 14**), the influence of pine species on the amounts of nitrogen (N), potassium (K), and phosphorus (P) was significant at P<0.05, while the amounts of zinc (Zn) and iron (Fe) were significant at P<0.01. In addition, the effect of irrigation levels on nutrient content was statistically significant (P<0.01) (**Table 14**). The *P. mugo* had the highest concentrations of the nutrients N, K, and Fe (**Table 15**). Additionally, *P. banksiana* population 8310055 had the highest P element content, whereas *P. nigra* had the highest Zn content (**Table 15**). The lowest nutrient content was found in *P. banksiana*, population 8960049. (**Table 15**). Under extreme stress, the nutritional content of N, P, K, Fe, and Zn reduced by 43%, 15%, 9%, 52%, and 20%, respectively (**Table 16**). The interaction between drought stress and pine species treatments for P content revealed that *P. banksiana* population 8310055 under normal irrigation had the highest *P content*, whereas *P. banksiana* population 8960049 under intense drought stress had the lowest P content (**Table 7**).

**Table 14:** Analysis of variance for the content of shoot nutrient (N, P, K, Fe, and Zn) of five pine species (*P. brutia* var. eldarica, *P. mugo, P. nigra, P. banksiana* 1*, P. banksiana* 2) under three drought stress treatments (normal, mild, and intense drought stress).

S.V	df	Mean square				
		Nitrogen (N)	Phosphorus (P)	Potassium (K)	Iron (Fe)	Zinc (Zn)
Rep	2	0.0043^ns^	0.0002^ns^	0.14^ns^	6.8^ns^	0.097^ns^
Species	4	0.032^*^	0.002^*^	0.2 ^*^	72^**^	0.259^**^
Stress	2	1.86^**^	0.166^**^	8.89^**^	2675^**^	23.32^**^
Species × Stress	8	0.002^ns^	0.392^**^	0.58^ns^	3.85 ^ns^	0.0047 ^ns^
Error	28	0.011	6.22	0.07	5.75	0.059

*: Significant at the 0.05 probability level, **: Significant at the 0.01 probability level, ns, non-significant.

S.V, source of variance; df, degree of freedom; Rep, replication; CV, coefficient of variance.

Normal: 100% field capacity (FC), Mild: 75% FC, Intense: 50% FC.

**Table 15:** Mean comparison of shoot nutrient content (N, P, K, Fe, and Zn) for five pine species (*P. brutia* var. eldarica, *P. mugo, P. nigra, P. banksiana* 1*, P. banksiana* 2).

Species	Nitrogen (N) (%)	Phosphorus (P) (mg. g^-1^)	Potassium (K) (mg. g^-1^)	Iron (Fe) (mg. kg^-1^)	Zinc (Zn) (mg. kg^-1^)
*P. brutia.* var. eldarica	1. 32 ± 0.29^ab^	1.23 ± 0.27^ab^	16.52 ± 1.38^a^	39.65 ± 11.27^ab^	11.41 ± 1.017^ab^
*P. mugo*	1.386 ± 0.29^a^	1.21 ± 0.29^abc^	16.51 ± 1.55^a^	41.80 ± 10.93	11.26 ± 0.90^a b^
*P. nigra*	1.30 ± 0.28^ab^	1.18 ± 0.28^c^	16.35 ± 1.39^ab^	37.85 ± 11.02^bc^	11.64 ± 1.17^a^
*P. banksiana*1	1.25 ± 0.29^b^	1.24 ± 0.26 ^a^	16.29 ± 1.44^b^	36.64 ± 11.64^cd^	11.28 ± 0.97^b^
*P. banksiana*2	1.25 ± 0.30^b^	1.20 ± 0.27^bc^	16.17 ± 1.44^b^	34.37 ± 12.56^d^	11.23 ± 1.02^b^

In each column, means followed by a common letter are not significantly different according to the LSD test at an alpha level of 0.05.

P. banksiana1: P. banksiana population 8310055, P. banksiana2: P. banksiana population 8960049.

**Table 16:** Mean comparison of shoot nutrient content (N, P, K, Fe, and Zn) under three drought stress treatments (normal, mild, and intense drought stress).

Drought stress levels	Nitrogen (N) (%)	Phosphorus (P) (mg. g^-1^)	Potassium (K) (mg. g^-1^)	Iron (Fe) (mg. kg^-1^)	Zinc (Zn) (mg. kg^-1^)
Normal	1.65 ± 0.07^a^	1.32 ± 0.08^c^	17.07 ± 0.19^a^	49.52 ± 1.63^a^	12.69 ± 0.27^a^
Mild drought stress	1.30 ± 0.03^b^	1.21 ± 0.04^b^	16.49 ± 0.26^b^	41.27 ± 3.10^b^	11.20 ± 0.12^b^
Intense drought stress	0.94 ± 0.10^c^	1.11 ± 0.06^a^	15.55 ± 0.14^c^	23.40 ± 2.85 ^c^	10.21 ± 0.10^c^

In each column, means followed by a common letter are not significantly different according to the LSD test at an alpha level of 0.05.

Normal: 100% field capacity (FC), Mild: 75% FC, Intense: 50% FC.

### 3.9 Visual quality

According to the analysis of variance (**Table 6**), the influence of pine species and irrigation levels on visual quality was significant (P<0.01). The *P. mugo* and *P. brutia* var. eldarica species displayed the best visual quality, and population 8960049 of the *P. banksiana* species displayed the lowest visual quality, according to the mean comparison for visual quality (**Table 7**). In comparison to the normal treatment, intense drought stress led to a 40% decrease in visual quality. (**Table 8**).

### 3.10 Relationship between traits

**Figure 1:**
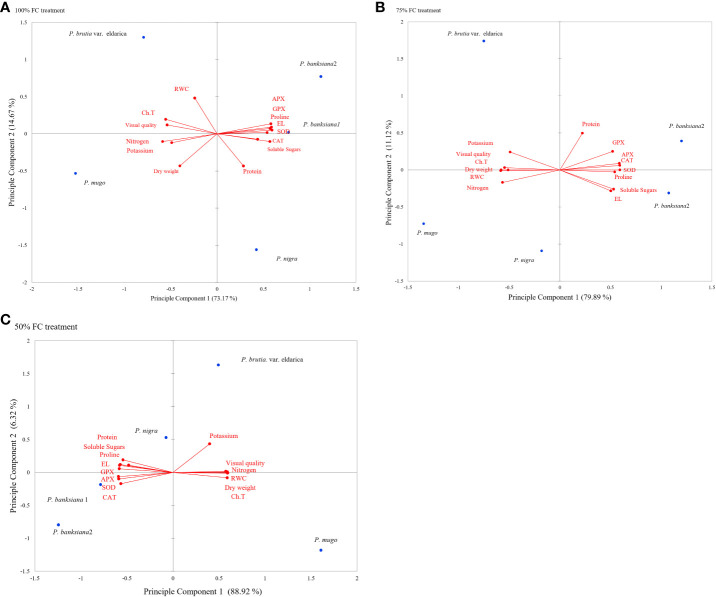
**(A-C)** Principal component analysis (PCA) for physiological and morphological traits under drought stress treatments (100 FC, 75% FC, 50% FC) and five pine species (P brutia var eldarica, P. mugo, P. nigra, P. banksiana 1, P. banksiana 2). P. banksiana 1: P. banksiana population 8310055, P. banksiana 2: P. banksiana population 89600-49 100% FC normal, 75% FC mild stress, 50% FC: intense stress Ch.T, total Chlorophyll; RWC, relative water content; EL, electrolyte leakage; CAT, catalase; GPX, guaiacol peroxidase; APX, ascorbate peroxidase; SOD, superoxide dismutase; N, nitrogen; K, potassium.

Principal component analysis (PCA) was used to evaluate the responses and choose the best genotypes for the three irrigation treatments by looking at the general association between attributes and genotypes (**Figure 1**). The degree of connection between features is shown by the cosine of the angles between the vectors. In the biplot of PCA, the angles between vectors of traits (variables) show the approximate value of correlation between traits. In the biplot of PCA, vectors of traits (variables) showing acute angle are positively correlated (angle 90° to 0° in measure), whereas those formed obtuse or straight angles are negatively correlated (angle 90° to 180° in measure), and those with right angle have no correlation (angle 90° in measure). The strength of correlations increases for angles close to 0° and 180°, and the length of the vectors leading from a trait to its origin reveals the degree of variability and contribution of each trait in PCA. According to PCA data, the first two components (PC1 and PC2) explained more than 86%, 91%, and 95% of all genetic alterations under normal, mild, and intense drought stress, respectively (**Figure 1**).

Traits were found to be divided into three groups using PCA under normal irrigation treatment. Proline, EL, soluble sugars, and antioxidant enzymes (CAT, APX, GPX, SOD) are included in the first category. Additionally, there was a negative association between these characteristics and visual quality, DW, RWC, total chlorophyll, and content of nutrients (N and K). Protein characteristics were assigned to the second group and were favorably linked with proline, EL, soluble sugars, and antioxidant enzyme activity. Visual quality, DW, RWC, and total chlorophyll, N, and K were positively correlated in the third group. While PC1’s correlations for visual quality, DW, RWC, total chlorophyll, N, and K were all negative, it had positive coefficients for the activity of antioxidant enzymes, protein, proline, EL, and soluble sugars. *P. mugo* species had the maximum DW, visual quality, RWC, N, K, and total chlorophyll, according to the PCA results for normal irrigation. The antioxidant enzyme activity, soluble sugars, proline, and EL content were higher in populations 8310055 and 8960049 of the *P. banksiana* species. Furthermore, population 8310055 of the *P. banksiana* species displayed higher EL, proline, and soluble sugars content, while population 8960049 had higher antioxidant enzyme activity and protein content (**Figure 1A**).

Three categories of traits were identified by the PCA results for mild drought stress. The traits in the first group had a positive correlation and included CAT, APX, GPX, SOD, proline, EL, and soluble sugars. Visual quality, total chlorophyll, RWC, N, K, and DW all showed negative correlations with these characteristics. Protein content made comprised the second group, which was positively associated with the first. Visual quality, total chlorophyll, RWC, N, K, and DW made comprised the third group, and these traits had a positive correlation with it while having a negative correlation with the first group. PC1 demonstrated positive coefficients for antioxidant enzyme activity, protein, proline, EL, and soluble sugars, according to the findings. It had negative effects on visual quality, DW, RWC, N, K, and total chlorophyll, in contrast. The findings of PCA for the species indicated that *P. mugo* had the highest levels of total chlorophyll, DW, RWC, N, K, and visual quality under mild stress circumstances (**Figure 1B**).

The PCA analysis revealed a positive correlation between visual quality, total chlorophyll, RWC, N, K, and DW attributes when the plant is experiencing intense drought stress. Antioxidant enzyme activity, proline, EL, soluble sugars, and protein all had a negative correlation with these characteristics. The DW, visual quality, RWC, N, K, and total chlorophyll all displayed positive correlations for PC1, while antioxidant enzyme activity, soluble sugars, EL, proline, and protein all displayed negative coefficients (**Figure 1C**).

Additionally, the data demonstrated that the trend of the results remained unchanged when the contents of protein, soluble sugar, carotenoid, proline, and chlorophyll a, b, total, and dry weight were calculated. However, as drought stress increased, the contents of proline and soluble sugar increased and those of photosynthetic pigments and protein decreased (**Table 17**). Furthermore, the findings showed that *P. mugo* had the greatest total, carotenoid, and chlorophyll a, b, content. The population of *P. banksiana* species population 8960049, on the other hand, has the lowest levels of total, carotenoid, and chlorophyll a and b (**Table 17**). Population 8310055 of *P. banksiana* had the highest proline and soluble sugar concentrations, while *P. mugo* had the lowest, according to the mean comparison for these two parameters. *P. banksiana* species population 8960049 had the highest protein content, whereas *P. mugo* species had the lowest, according to the mean comparison of protein for species (**Table 17**).

**Table 17:** Mean comparison of chlorophyll a, b, total, carotenoid, proline, soluble sugars, and protein for five pine species (*P. brutia* var. eldarica, *P. mugo, P. nigra, P. banksiana* 1*, P. banksiana* 2) under three drought stress treatments (normal, mild, and intense drought stress) based on dry weight.

Treatment		Chlorophyll a (mg.gr^-1^ dw)	Chlorophyll b (mg.gr^-1^ dw)	Chlorophyll Total (mg.gr^-1^ dw)	Carotenoid (mg.gr^-1^ dw)	Proline (mg.gr^-1^ dw)	Soluble sugars (mg.gr^-1^ dw)	Protein (mg/gr^-1^dw)
Species	*P*. eldarica	0.962 ± 0.19^b^	0.381 ± 0.08^a^	1.333 ± 0.27^b^	0.447 ± 0.09^a^	3.43 ± 1.18^d^	56.62 ± 6.44^bc^	2.27 ± 0.31^b^
	*P. mugo*	1.010 ± 0.16^a^	0.388 ± 0.06^a^	1.390 ± 0.22^a^	0.454 ± 0.09^a^	3.17 ± 1.18^e^	55.38 ± 7.72^c^	2.11 ± 0.17^c^
	*P. nigra*	0.941 ± 0.19^bc^	0.329 ± 0.05^b^	1.270 ± 024^c^	0.413 ± 0.1^c^	3.82 ± 1.11^c^	59.36 ± 5.97^ab^	2.40 ± 0.27^ab^
	*P. banksiana*1	0.931 ± 0.21^c^	0.332 ± 0.06^b^	1.221 ± 0.27^c^	0.429 ± 0.11^c^	4.06 ± 1.19^a^	61.34 ± 5.93^a^	2.39 ± 0.29^ab^
	*P. banksiana2*	0.902 ± 0.22^d^	0.311 ± 0.07^c^	1.221 ± 0.29^c^	0.432 ± 0.11^c^	3.99 ± 1.21^a^	58.56 ± 6.72^bc^	2.44 ± 0.28^a^
Drought stress	Normal	1.033 ± 0.05^a^	0.378 ± 0.04^a^	1.411 ± 0.07^a^	0.499 ± 0.03^a^	2.04 ± 0.27^c^	44.59 ± 2.17^c^	100.23 ± 0.08^a^
	Mild	1.021 ± 0.07^a^	0.36 ± 0.06^a^	1.384 ± 0.12^a^	0.446 ± 0.03^b^	3.84 ± 0.40^b^	60.82 ± 2.59^b^	100.26 ± 0.19^a^
	Intense	0.78 ± 010^b^	0.30 ± 0.04^b^	1.07 ± 0.14^b^	0.35 ± 0.05^c^	5.71 ± 0.39^a^	72.48 ± 2.80^a^	99.42 ± 0.22^ab^

In each column, means followed by a common letter are not significantly different according to the LSD test at an alpha level of 0.05.

P. banksiana1: P. banksiana population 8310055, P. banksiana2: P. banksiana population 8960049.

Normal: 100% field capacity (FC), Mild: 75% FC, Intense: 50% FC.

## 4 Discussion

This study revealed that drought stress negatively affected the development of all pine species, as both the FW and DW of the shoots decreased significantly (**Table 4**). It appears that the restriction of cell formation and growth mediated by the reduction in turgor pressure accounts for the decrease in fresh weight of plants in dry conditions (Deligoz and Gur, 2015). The amount of dry matter in the aerial parts gradually decreases as a result of the soil’s ongoing loss of water, which reduces the size and surface area of the leaves (Bahreininejad et al., 2013). Similarly, Yin et al. (2018) showed that severe drought stress reduced the fresh and dry weights of *P. sylvestris* var. mongolica seedlings (Yin et al., 2018).

This experiment demonstrated that drought stress decreased leaf chlorophyll content. The effect of drought stress on the absorption of nutrients such as N and Fe, which play an essential role in the structure of chloroplasts, also contributes to the decrease in chlorophyll concentration. As a result of the decreased absorption of these ions, chlorophyll production decreases.

Our results are consistent with the majority of other available studies that reported the chlorophyll pigment decreased under intense drought in *P. densata* (Gao et al., 2009), *Picea abies* (Ditmarová et al., 2010), *P. halepensis* (Baquedano and Castillo, 2007), *P. Massoniana* (Xie et al., 2019), *P. nigra* Arn. and *P. brutia* Ten. (Deligöz and Cankara, 2020), and *Cupressus arizonica* G. (Aalipour et al., 2023). This study also revealed that resistant genotypes have higher levels of carotenoids and chlorophyll (**Table 3**), which likely contributed to a more effective defensive system that preserves higher visual quality (**Table 7**). Carotenoids have a protective impact against oxidative stress, contribute to chlorophyll detoxification, and mitigate the toxicity of free radicals (Zhang et al., 2021). Plants with a higher carotenoid content have a more effective defense against reactive oxygen species and exhibit greater water stress tolerance (Noctor et al., 2016; Aalipour et al., 2023).

Our research demonstrated that RWC dropped when faced with the stress of a water shortage (**Table 8**). Examining RWC is crucial when selecting stress-tolerant plants, as a higher RWC under stressful conditions indicates increased water uptake and cell turgescence. The presence of the osmotic regulation mechanism protects the tissue’s structural and functional properties. Numerous studies have noted that under stress, the relative water content decreases (Amiri et al., 2017; Nxele et al., 2017; Shivakrishna et al., 2018).

The first thing that happens when plants are stressed by drought is that their relative water content goes down and their stomata close. This stops the production of photosynthetic components and causes a loss in yield (Torabian et al., 2018). Bhardwaj et al. (2018) showed that when a plant is subjected to water deficit stress, the amount of tissue water falls and the relative water content of the leaf decreases (Bhardwaj et al., 2018). Consequently, the higher the relative leaf water content of a cultivar under drought stress circumstances, the greater its drought resistance.

According to the results of this experiment, *P. mugo* and *P. brutia* var. eldarica species likely maintained their RWC under stress conditions by properly adjusting their osmosis (**Table 7**). Cell turgor and the plant’s osmotic potential are both directly correlated with the water potential of the plant. The rate of photosynthesis and the production of dry matter also are accelerated by raising the RWC because it causes the stomata to open more quickly (Deshpande et al., 2021).

The results of PCA for the species indicated that drought tolerance might be further increased by choosing resistant species based on DW, RWC, and total chlorophyll (**Figure 1C**). Under severe stress conditions, *P. mugo* species demonstrated more resistance and displayed the highest DW, total chlorophyll, RWC, N, K, and visual quality (**Figure 1C**).

In this investigation with increasing levels of drought stress, the rate of electrolyte leakage increased (**Table 8**). Drought stress reduces the integrity of the cell membrane and causes electrolytes and intracellular chemicals to be released. According to Cesari et al. (2016), the fatty acids in the cell membrane have a significant impact on the fluidity of the membrane, and dehydration stress causes a shift in membrane fluidity followed by an increase in EL. (Cesari et al., 2016).

Unsaturated fatty acids rise as a result of several alterations in membrane phospholipids brought on by a water deficit. When under extreme stress, the membrane’s bilayer phospholipids undergo hexagonal transformation, the membrane structure becomes porous, and material leakage occurs (Wang et al., 2012). The ROS produced by electrolyte leakage in plants lead to oxidative stress, which destroys cell membranes and other biological components found in living cells When under drought stress, cell membrane integrity is maintained, indicating that there are drought-tolerance regulatory systems (Duan et al., 2005). This study showed that *P. brutia* var. eldarica and *P. mugo* species are more resistant to drought stress than populations of *P. banksiana* species and *P. nigra* and had membrane stability to those species (**Table 7**).

The overall response of pine seedlings to water stress situations as an osmotic regulator was likely proline accumulation under severe drought conditions. *P. mugo* seedlings had less proline than seedlings of other species, indicating that this species is less susceptible to the effects of osmotic stress. Under extreme stress, seedlings store proline in their tissues because it is involved in osmotic control; the quickest increase happens in the leaves (Li et al., 2021). Raising proline content in drought-stressed plants is an adaptation for overcoming stress. Proline can perform a variety of tasks under stressful circumstances, including generating osmotic interactions, safeguarding protein and cell membrane structures, stabilizing intracellular structures, and eliminating free radicals (Shafi et al., 2019).

Proline accumulates to distribute water into the cell when there is a water shortage because proline production from glutamic acid frequently takes place in the cytosol and chloroplast of plant cells (Verslues and Sharma, 2010). Proline is delivered to organs under control conditions, particularly vacuoles and plasmids, and when the plant is affected by drought, proline is moved from the vacuoles to the cytosols (Sancho-Knapik et al., 2017).

The results also showed that soluble sugars content rose with increasing drought stress (**Table 8**), and population 8310055 of the *P. banksiana* species showed greater content than the other populations (**Table 7**). The ongoing consumption of carbohydrates produced at the plant’s growth sites is largely responsible for the reduced carbohydrate content of the leaf under normal conditions. Extreme stress causes an increase in total soluble carbohydrates because the plant boosts its internal osmotic pressure in order to absorb soil nutrients and water. The distribution of hydrocarbons is influenced both directly and indirectly by environmental conditions such as water deficiency and plant hormones. The buildup of organic molecules such as carbohydrates and amino acids in the cytoplasm of plants plays a crucial role in regulating osmotic pressure. (Hajiboland et al., 2017). Additionally, maintaining water-soluble carbohydrates preserves cell membranes, delays protein deterioration, maintains the turgor of leaves, and keeps the plant from decaying by giving it the energy it requires (Deng et al., 2019). According to earlier research on *P. massoniana* (Shao et al., 2022) and *C. arizonica* G. (Aalipour et al., 2020), plants primarily store soluble sugars to improve osmotic adjustment during stress.

Our investigation’s findings showed that the stress of drought elevated the activity of antioxidant enzymes (**Table 12**). According to Kormuťák et al. (2019), antioxidant systems in plants function as a crucial defense mechanism against drought stress that enhances plant tolerance to environmental challenges. In the current experiment, extreme drought stress boosted antioxidant activity among several pine species, with population 8960049 of *P. banksaina* species having the highest degree of antioxidant enzyme activity. In this process, the SOD plays a critical role in shielding cells from the harmful effects of superoxide radicals produced in diverse cell sections by catalyzing the conversion of superoxide (O_2_^-^) radicals to H_2_O_2_ and O_2_ (McKersie et al., 1993). As an enzyme separating agent, the hydrogen peroxide transformed into water and oxygen molecules by the enzymes CAT, GPX, and APX. Zhou et al. (2021), reported that the activity of antioxidant enzymes increased under drought stress in *P. tabulaeformis* (Zhou et al., 2021).

Both populations of the *P. banksiana* species displayed higher levels of antioxidant enzyme activity, soluble sugars, proline, and protein under control irrigation conditions. For control irrigation treatment, these species can be chosen and cultivated (**Figure 1A**). Both populations of the *P. banksiana* species responded to mild drought stress conditions similarly to typical circumstances (**Figure 1B**). In this study, based on the PCA analysis, we discovered a positive correlation between antioxidant enzymes, protein, EL, soluble sugars, and proline activity in drought-sensitive species (*P. banksiana* population 8960049) (**Figure 1C**).

In this experiment, nitrogen, potassium, iron, and zinc were taken up less than usual (**Table 16**) when the plants were under intense drought stress. The highest concentrations of N and Fe were found in *P. mugo* species. Population 8310055 of the *P. banksiana* species had the highest P concentration, *P. nigra* had the highest Zn concentration, and *P. brutia* var. eldarica had the highest K concentration (**Table 15**). Population 8960049 of the *P. banksiana* species showed the lowest concentrations of N, K, P, Fe, and Zn (**Table 15**).

In conditions of drought stress, the absorption of nutrients, particularly nitrogen, is reduced for a number of reasons, including the restriction of root growth and expansion, the reduction of the root’s ability to absorb nutrients due to the low osmotic potential around the root, and the prevention of the transfer of substances and nutrients from the root to the aerial part of the plant (Bista et al., 2018).

Chtouki et al. (2022) reported that as soil moisture increases, the absorption of micronutrients such as iron, zinc, and phosphorus increases because the absorption of these nutrients is strongly related to the quantity of moisture accessible to the plant (Chtouki et al., 2022). These results are similar to the findings of this study (**Table 16**). The decrease in mass flow of water produced by the stress of water scarcity disrupts the plant’s ability to absorb nutrients. Due to a shortage of water, the mobility and availability of phosphorus in the soil and, consequently, the plant’s ability to absorb phosphorus are diminished, resulting in a drop in phosphorus concentration and absorption under drought stress conditions (Tariq et al., 2018). In this study, drought stress lowered plant phosphorus absorption due to the limited mobility of phosphorus (**Table 16**). Population 8960049 of the *P. banksiana* and *P. mugo* species had the lowest P concentration, and population 8310055 of the *P. banksiana* had the highest P concentration (**Table 18**).

**Table 18:** Interaction effect of drought stress treatments (normal, mild, and intense drought stress) on five pine species (*P. brutia* var. eldarica, *P. mugo, P. nigra, P. banksiana* 1*, P. banksiana* 2), for phosphorus nutrient content.

Phosphorus (mg. g^-1^)	Species	Drought stress levels
Normal	*P. brutia.* var. eldarica	1.55 ± 0.04^b^
	*P*. *mugo*	1.26 ± 0.07^de^
	*P*. *nigra*	0.88 ± 0.05^fg^
	*P*. *banksiana*1	1.64 ± 0.02^a^
	*P*. *banksiana*2	1.3 ± 0.10
Mild drought stress	*P. brutia.* var. eldarica	0.92 ± 0.03^f^
	*P*. *mugo*	1.55 ± 0.05^b^
	*P*. *nigra*	1.24 ± 0.04^e^
	*P*. *banksiana*1	0.86 ± 0.03^g^
	*P*. *banksiana*2	1.48 ± 0.04^c^
Intense drought stress	*P. brutia.* var. eldarica	1.21 ± 0.07^e^
	*P*. *mugo*	0.84 ± 0.07^g^
	*P*. *nigra*	1.47 ± 0.03^c^
	*P*. *banksiana*1	1.22 ± 0.02^e^
	*P*. *banksiana*2	0.84 ± 0.10^g^

In each column, means followed by a common letter are not significantly different according to the LSD test at an alpha level of 0.05.

P. banksiana1: P. banksiana population 8310055, P. banksiana2: P. banksiana population 8960049.

Normal: 100% field capacity (FC), Mild: 75% FC, Intense: 50% FC.

The decrease in potassium absorption under drought stress is attributable to the decrease in potassium mobility in the soil, the drop in transpiration rate, the reduction in root hydraulic conductivity, and the disruption in root membrane activity (Zia et al., 2021). In addition, under drought stress conditions, zinc and iron micronutrients are inaccessible to the plant and their amounts are drastically diminished (Choukri et al., 2020). Lamhamedi et al. (1992) reported that drought stress reduces N, P, and K concentrations in *P. pinaster* (Lamhamedi et al., 1992). The concentrations of N, K, P, Fe, and Zn in *C. arizonica* decreased during drought stress, according to a study (Aalipour et al., 2020).

This study showed that different pine species’ aerial parts had lower total protein contents due to drought stress (**Tables 11**, **12**). Due to increased protease enzyme activity under severe drought conditions, protein synthesis declines (Liang et al., 2018). Additionally, an increase in proline content that maintains osmotic control under water deficit conditions is linked to a decrease in protein content (Chun et al., 2018). Protein levels sharply dropping is a sign of a species’ vulnerability to drought stress (Turco et al., 2004). So, both the degradation of protein and the decrease in its synthesis can be considered the causes of the significant increase in proline concentration as well as the significant drop in protein in pine leaves.

This experiment investigated how severe drought stress affected several pine species’ aesthetic appeal and how it induced more yellow and necrotic leaves (**Tables 7**, **8**). The plant’s color and green index diminish under water deficit stress due to chlorophyll degradation, which greatly lowers the visual quality (Lagomarsino et al., 2021).

## 5 Conclusions

The findings of this research revealed that intense drought stress decreased pine seedling growth parameters and promoted deterioration. However, pine seedlings of different species deployed osmotic adjustment mechanisms, such as soluble sugars and proline production, to preserve water content under both mild and intense drought stress. The study also found that intense drought stress caused serious damage to all species, whereas mild drought stress was manageable and only slightly harmful to pines’ growth and physiological properties. Furthermore, the study highlighted the importance of plant physiological processes in screening for drought resistance. RWC, osmotic adjustment, photosynthetic efficiency, and antioxidant defense mechanisms were crucial parameters for identifying drought-resistant genotypes. PCA analysis studies revealed that imposing drought stress alters the connection between characteristics. In addition, our study found that different species respond to drought stress in different ways due to possible genetic differences. Therefore, superior genotypes could be chosen with a focus on the PC1 component by following traits such as DW, Chl. T, RWC, N, K, and visual quality. For example, *P. mugo* maintained its photosynthetic structure under drought stress conditions by retaining the proper RWC and chlorophyll pigment, and N absorption, resulting in improved visual quality. In conclusion, based on the morphological and physiological properties of pine cultivars, the study identified *P. mugo* and *P. brutia* var. aldarica as appropriate species for planting in dry and low-water environments, while *P. banksiana* was found to be susceptible to drought.

## Data availability statement

The raw data supporting the conclusions of this article will be made available by the authors, without undue reservation.

## Author contributions

KN: Methodology, Data curation, Investigation, Software, Visualization, Writing – original draft. AN: Conceptualization, Project administration, Supervision, Writing – review & editing. MH: Supervision, Writing – review & editing. NE: Writing – review & editing. MR: Writing – review & editing, Funding acquisition, Methodology. AS: Data curation, Formal Analysis, Funding acquisition, Writing – review & editing.
